# Surgical Management of the Metastatic Spine Disease: A Review of the Literature and Proposed Algorithm

**DOI:** 10.1177/21925682221146741

**Published:** 2022-12-14

**Authors:** Humaid Al Farii, Ahmed Aoude, Ahmed Al Shammasi, Jeremy Reynolds, Michael Weber

**Affiliations:** 1 Division of Orthopaedic Surgery, 5620 McGill University, Montreal, QC, Canada

**Keywords:** cervical, lumbar, thoracic, compression, decompression, tumors, metastases, and oncology

## Abstract

**Study Design:**

Narrative Review. The spine remains the most common site for bony metastasis. It is estimated that up to 70% of cancer patients harbor secondary spinal disease. And up to 10% will develop a clinically significant lesion. The last two decades have seen a substantial leap forward in the advancements of the management of spinal metastases. What once was a death sentence is now a manageable, even potentially treatable condition. With marked advancements in the surgical treatment and post-operative radiotherapy, a standardized approach to stratify and manage these patients is both prudent and now feasible.

**Objectives:**

This article looks to examine the best available evidence in the stratification and surgical management of patients with spinal metastases. So the aim of this review is to offer a standardized approach for surgical management and surgical planning of patients with spinal metastases.

## Introduction

The spine remains the most common site for bony metastases.^1^ It is estimated that up to 70% of cancer patients harbor secondary spinal disease^2^ and up to 10% will develop a clinically significant lesion.^3^ Even among the pediatric age group, the prevalence of metastatic spine tumors has increased from 88.5 to 117.9 per 100 000 within 10 years from 2003 to 2012,^4^ and that is not surprising as the World Health Organization estimates the incidence of cancer to increase 1.5 times by 2020 from the 10 million diagnosed in 2000 worldwide.^5^

The last two decades have seen a substantial leap forward in the advancements of the management of spinal metastases. What once was a thought to be non-curable disease is now a manageable, even potentially curable condition. The goal of surgery is to stabilize a mechanically unstable spine, decompress spinal cord compression, remove epidural disease to allow spine stereotactic radiosurgery (SRS) or spine stereotactic body radiotherapy (SBRT) treatment, establish a histological diagnosis, and to provide local control when radiotherapy cannot be safely delivered.

With the advancement of surgical techniques in spinal surgery, the surgical management of spinal metastases is often no longer a simple decompression, which has proven itself to be insufficient in the vast number of patients. Present day surgical management, when appropriately planned, can improve prognosis, maintain and/or recover neurological function or ambulation status, provide effective pain control and improve quality of life.^6^ Cancer staging requires complete clinical history, physical examination, laboratory and imaging investigations. A standardized approach to the management of patients with spine metastases is of profound importance. In turn, this will ensure that all newly diagnosed cases of spinal metastases will receive an evidence-based approach and standardized clinical care pathway including the referral for surgical evaluation of these cases by specialists of different medical disciplines, given the management of these patients is almost always multidisciplinary.

This article looks to examine the best available evidence in the stratification and surgical management of patients with spinal metastases. The aim of this article is to offer a standardized approach for surgical management and surgical planning of patients with spinal metastases.

## Prognostic Stratification

Historically patients with spinal metastases were managed by posterior decompression alone. Results were poor in terms of regaining or retaining ambulatory status and case series were plagued by high complication rates.^7,8^ Surgical intervention for these patients was largely abandoned in favor of conventional Radiotherapy based on superiority studies in terms of neurological function improvement and pain control.^9-17^ Several case reports published subsequently showed better outcome with anterior or anterolateral surgery.^18-22^ It was met with initial skepticism, with concerns that results may have been attributed to patient selection bias. It was not until Patchell et al, multicenter randomized clinical trial that reported on an improved outcome of direct decompressive surgical resection with radiotherapy vs radiotherapy alone, that surgery again became popularized as the standard treatment for spinal metastases.^23^ Following this, a need emerged for proper patient selection to insure optimum outcomes. Several prognostic scoring systems have been developed and modified over recent years to aid surgeons in patient selection for surgery. Most of these scoring systems are based on: functional capacity at the time of diagnosis, neurological status, the tumor type, and the spinal, skeletal and visceral tumor load.^24-26^ Pre-operative work up aims at addressing these questions.

**Figure 1.**
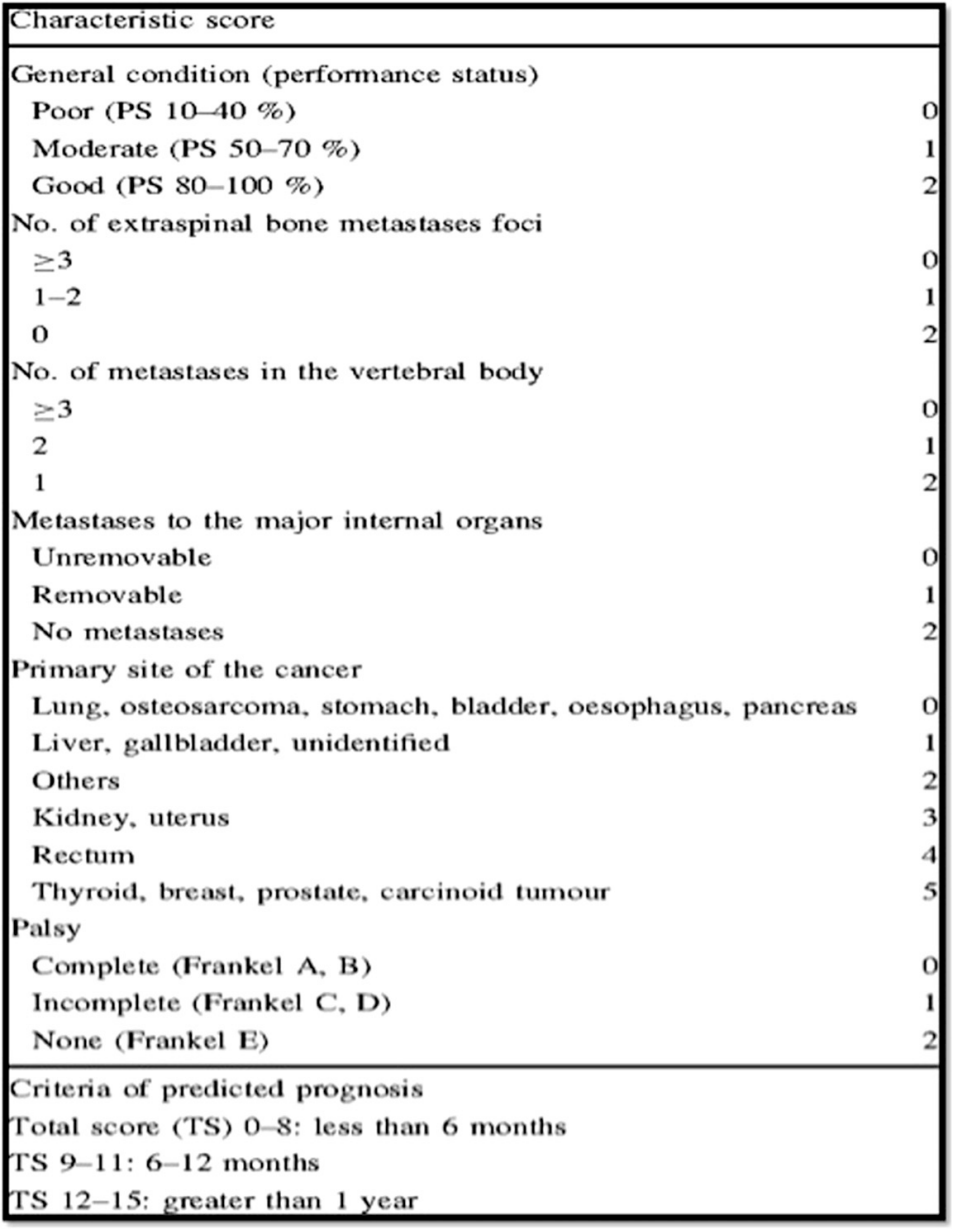
Revised Tokuhashi score published in 2005. Overall score is calculated based on 6 prognostic variables.

Tokuhashi et al reported their “Scoring system for preoperative evaluation of a patient’s prognosis with metastatic spinal tumor” in 1989.^24^ Retrospective data was collected from 64 patients with spinal metastases and analyzed to develop a comprehensive scoring system. Six variables were identified to be significant in prognostic stratification of these patients (Figure 1). A revised version was published in 2005^25^ where the scores of the “Primary site of cancer” were modified. The overall score is calculated and patients are divided into 3 groups: Conservative management (score 0-8), Palliative surgery (score 9-11) and lastly Excisional surgery (score >12) with predicted survival reported at <6, >6 and >12 months respectively. The result of a follow up prospective study in which the management was selected according to this revised version was published in 2009 with reported 87.9% consistency between predicted and actual prognosis.^27^

**Figure 2.**
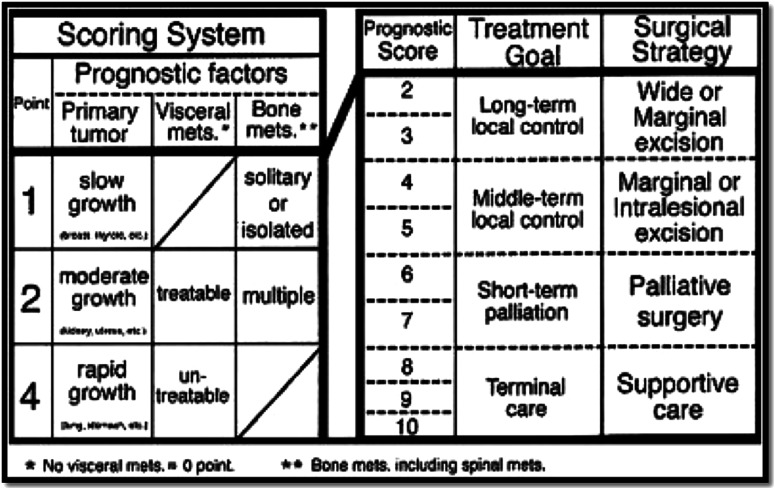
Tomita score published in 2001 offered a more simplified way to stratify patients based on 3 prognostic variables.

Tomita and Kawahara et al, retrospectively evaluated 67 patients treated for spinal metastases, including conservative management, between 1987 and 1991 and developed a new and simplified scoring system in 2001.^26^ Three factors were found to impact prognosis: the rate of growth of the primary tumor, the number of bone metastases and presence or absence of visceral metastases. The score of the three components are added together to produce an overall score ranging from 2-10, from good to poor prognosis respectively. Patients are stratified into 4 groups with suggested surgical treatment accordingly (Figure 2). The mean survival for each group were reported at 50, 23.5, 15 and <6 months. General performance status was not part of the Tomita scoring system while it was significant in Tokuhashi’s scoring system as well as others.^24,25,28-30^ Perhaps performance status may be just a mere reflection of the tumor load and presence of visceral metastases rather than being an independent prognostic factor.^26,31^

Several other prognostic scoring systems have been developed over the years, Baur score,^32^ Sioutos score,^28^ Van der Linden score,^29^ Katagari score^30^ amongst a few. However, Tokuhashi and Tomita scores remain the two most commonly used and studied.^33-41^ Several external validation studies of these two scoring systems have been published in the literature, both prospective and retrospective, with conflicting results.^31,33-38,42,43^ However, no scoring system had more than 90% consistency between the predicted and the actual survival time.^44^

It is somewhat expected that these scoring systems tend to underestimate the overall survival. Advancements in the surgery, radiotherapy and chemotherapy perhaps account for these findings.^45^ However, these scoring systems are devised to segregate patients into groups in order to offer the best management according to “predicted” survival. Novel tumor biomarkers and tumor epigenetics along with novel hormonal and immunotherapeutic will perhaps positively skew the survival curves even more given better disease control.^46-49^ Perhaps in the future we will see more tumor-specific scoring systems taking into account specific biological or epigenetic characteristics.^50-52^ Such advancements however should not affect the decision to offer surgical intervention in the majority of cases.

## Spine Instability

In the absence of Neurological compression, surgical intervention should be considered for restoration or maintenance of spinal stability. The concept of instability in spinal oncology received significant interest in the past decade. Perhaps the earliest attempt to systematically define spinal instability comes from Kostuik et al work.^53^ The spinal column was divided into 6 spinal segments. The spine was considered unstable if 3 or more segments are involved. Tomita et al considered the results of several previous studies^53-55^ and determined that instability is presumed if one of the following radiologic features is present: transitional deformity, vertebral body collapse greater than 50%, three column involvement (as defined by Denis^54^), involvement of the same column in two or more adjacent levels.^26^ Several important considerations are not addressed by these systems, such as: the lack of clinical input into the assessment, the pathological effect of the tumor on the vertebral integrity (osteolytic vs osteoblastic), the mobility of the segment involved. Such factors may be more important when determining the risk of impending instability.^56^

**Figure 3.**
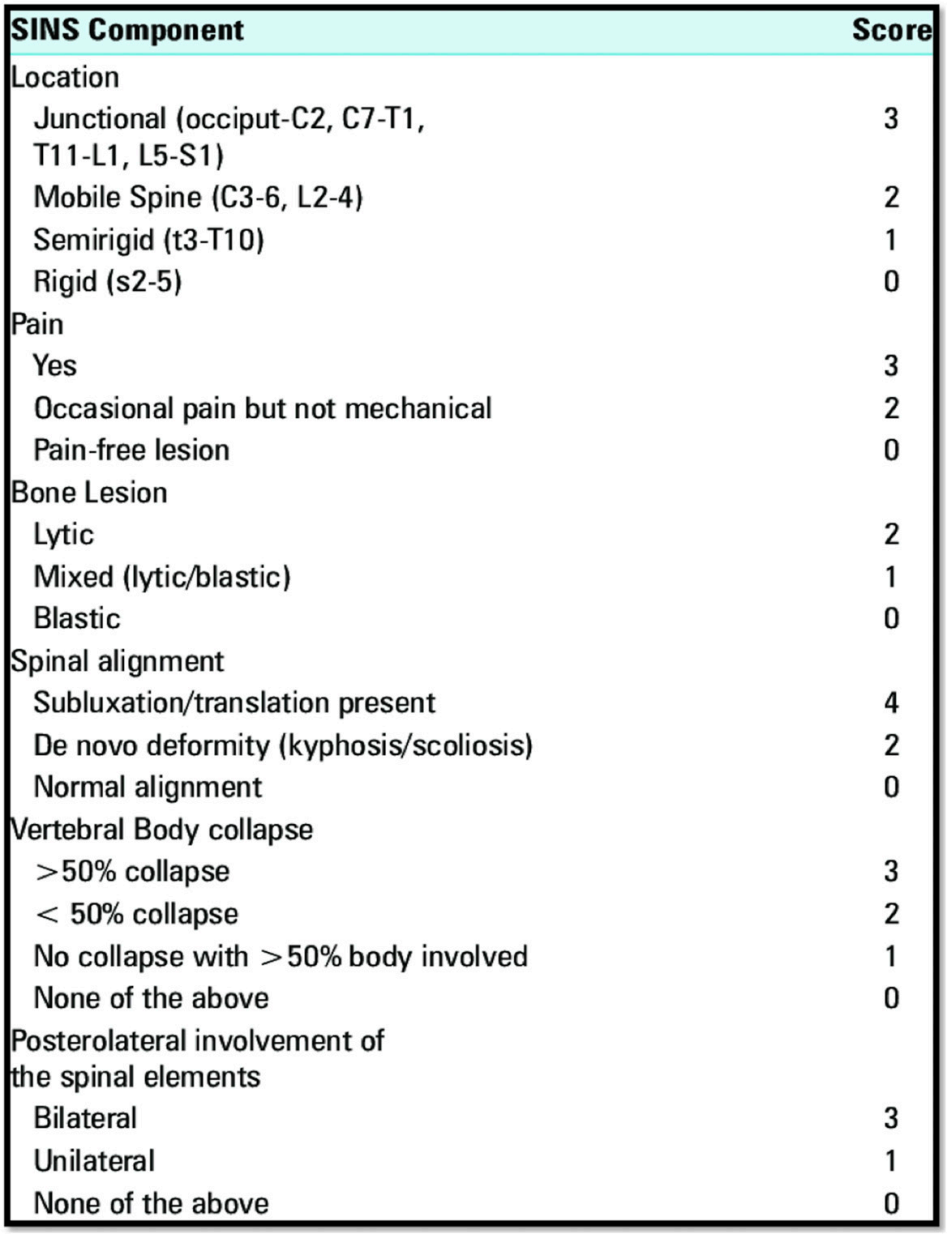
Spinal instability in Neoplastic Spine (SINS) score.

A relatively new scoring system encompasses most of the important aspects in the assessment of spinal stability. Fisher et al devised the “Spinal instability in Neoplastic Spine (SINS)” score in 2010^57^ (Figure 3). The affected segment is examined for several factors: the spinal level, the presence of mechanical or postural pain, the spinal alignment (contrasted supine and upright radiographs), bone lesion quality, vertebral body involvement and posterior elements involvements. Three categories yield: Stable (0-6), impending instability (7-12), Unstable (13-18). The Spine Oncology Study Group defines spinal instability as a “loss of spinal integrity as a result of a neoplastic process that is associated with movement-related pain, symptomatic or progressive deformity, and/or neural compromise under physiologic loads.”^57^

Fourney et al examined the inter- and intraobserver reliability of the SINS score in 30 patients and demonstrated near-perfect agreement for the three clinical categories mentioned.^58^ The sensitivity and specificity of the SINS score for potentially unstable or unstable lesions were 95.7% and 79.5% respectively. Fisher et al also tested the reliability of the SINS score among Radiation Oncologists and demonstrated substantial inter-observer and excellent intra-observer reliability.^59^ None of the unstable cases were scored stable by Radiation Oncologists, which would ensure appropriate referral of these cases for surgical consultation. A recent meta-analysis examine the reliability of the SINS System that showed highly reliable both within and across observers, and the degree of reliability seems to increase with increased clinical exposure to metastatic spine disease.^60^ The assessment of spinal stability should be part of the pre-operative surgical planning for patients undergoing palliative intervention in the presence or absence of neurological compression.

## Surgical Planning

Surgical objectives should be individualized for each patient according to predicted prognosis and the clinical presentation.^61^ Surgical objectives include: resection with the aim for disease control, decompression of neural structures, maintaining or restoring spinal stability, and pain relief. Witham et al^13^ conducted an extensive review to compare radiotherapy and surgery. Radiotherapy alone resulted in a mean neurological improvement rate of 36%. More extensive surgical procedures resulted in greater neurological improvements with rates of 42%, 64%, and 75% in laminectomy, laminectomy plus stabilization, and anterior corpectomy plus stabilization respectively. Unfortunately, surgical morbidity, which was in the range of 21-26% correlated positively with the extensiveness of surgical procedure^62^ and the use of preoperative radiotherapy.^63^ The lack of improvements in complication and mortality rates despite surgical advancements is likely due to these same advances allowing more aggressive resections and a more complex patient population.^64^ Our approach for standardization of surgical treatment follows guidelines and techniques established in the recent literature of last two decades.

### Excisional Surgery

#### A. En Bloc Spondylectomy

The main surgical objective in this arm is to achieve tumor removal with aim for cure given predicted extended survival. En bloc resection is of profound importance in this group to minimize seeding of tumor in the surgical bed. Resection is followed by spinal reconstruction and instrumentation accordingly depending on the approach; posterior, anterior or combined. Several surgical planning schemes have been described in the past.^26,65,66^ We suggest using the Tomita et al classification for planning given the best available evidence in literature.^26,65^

**Figure 4.**
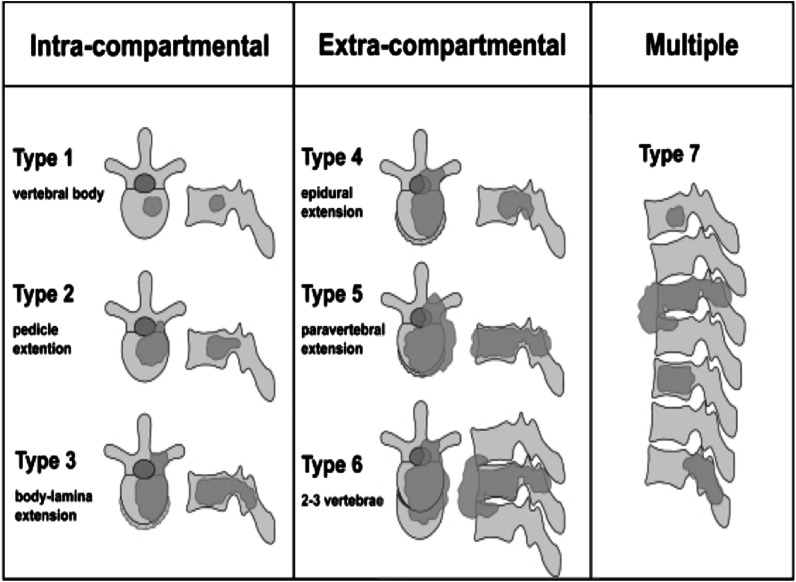
Extent of spinal involvement to be delineated on pre-operative imaging and would guide surgical planning. Tomita’s scheme is easy to apply and would help standardize surgical management.

In their work, Tomita et al proposed wide or marginal excision for long-term local control. There are seven types described according to extent of involvement^26^ (Figure 4). Types 1 through 3 involves cases where the tumor is within the vertebra “Intracompartmental”, depending on the extent of involvement (Type 1: anterior column, Type 2: extension to pedicle, Type 3: posterior column). Type 4 is when there is epidural extension with tumor at any site, while Type 5 is when there is paravertebral extension of the tumor. Type 6 is where 2-3 vertebrae are involved while type 7 indicates the presence of multiple lesions.

Wide or marginal resection is feasible for type 1 through 4 and selected cases of type 5. In some selected cases of type 5 lesions en bloc spondylectomy can be achieved if paraspinal extension can be removed safely. Stener et al,^67^ Roy-Camille et al^68^ and Tomita et al^65^ have all described techniques for single-stage complete spondylectomy using posterior approach. However, the lack of visualization of the ventral structures increases the risk for vascular injury. In addition these techniques are of limited applicability in the lumbar spine given the attachment of the psoas and iliacus muscles.^69^ Staged surgery is advisable in such cases where a first a posterior approach is done to remove the posterior spinal elements and perform cut across the pedicles on both sides. Spinal instrumentation provides the long-term stability needed. This is followed by a second stag anterior approach to remove the vertebral body and reconstruct the anterior column with titanium cage or bony strut such as autologous or cadaveric rib or fibular graft.

Interpreting the data available in the literature about total en bloc spondylectomy (TES) for spinal metastases should be done with caution. Several authors reported less than desirable outcomes; Cloyd et al systematically reviewed all cases of TES for both primary and metastatic spine lesions.^69^ The reported median time to recurrence was 2 years; the median overall survival was not reported. Similarly Sakaura et al reported their series of 12 patients treated with TES for metastases.^70^ None of the mentioned scoring systems were used however, the technique describe by Tomita et al was used. In their series, local recurrence occurred in 50% cases where paraspinal extension was present. Seven patients survived an average of 61 months however the other 5 died with mean survival of 23 months. When compared to reported outcome from world-renowned surgeons the difference is striking. Tomita et al prospectively followed 198 patients with metastatic tumors from 1989 to 2003, out of which 64 had TES. 43 patients of those had predicted long-term survival and 66.6% survived at 2 years, 46.6% survived 5 years. The median Kaplan-Meier survival was 3.5 years.^71^ Yao et al reported a case series of 40 patients treated with en bloc excision performed by Boriani S., Gokaslan Z.L. and Sundresan N. with median survival time longer than 3 years.^72^ Performing en bloc excision for spinal tumors is technically challenging. Furthermore, the term is perhaps used even when contaminated margins are present, or used interchangeably with gross total or radical resection and may not reflect the actual procedure performed.^70^ That could explain the 50% local recurrence when paraspinal extension was present in Sakaura et al series. We believe the term en bloc excision should only be used when the tumor is removed as a single specimen with no contamination of the surgical margin.

#### B. Separation Surgery and Stereotactic Radiosurgery

The suboptimal outcomes of invasive surgery and conventional radiotherapy in the treatment of spinal metastases have led many cancer centers to explore the potential of spine stereotactic body radiotherapy (SBRT) or spine stereotactic radiosurgery (SRS) as an alternative therapeutic option for this subset of patients.^73^ And the availability of SRS as one of the main radiotherapeutic modalities in certain centers has led to the development of “Separation surgery”.^74^ The term separation surgery was coined by Lilyana Angelov and Edward Benzel at The Cleveland Clinic, to designate a procedure in which tumour resection is limited to decompression of the spinal cord to create a gap to the tumour and provide a safe target for spine SRS. Such a technique helps to facilitate the delivery of an ablative dose to the residual tumour while sparing the spinal cord or cauda equinez.^75^

**Figure 5.**
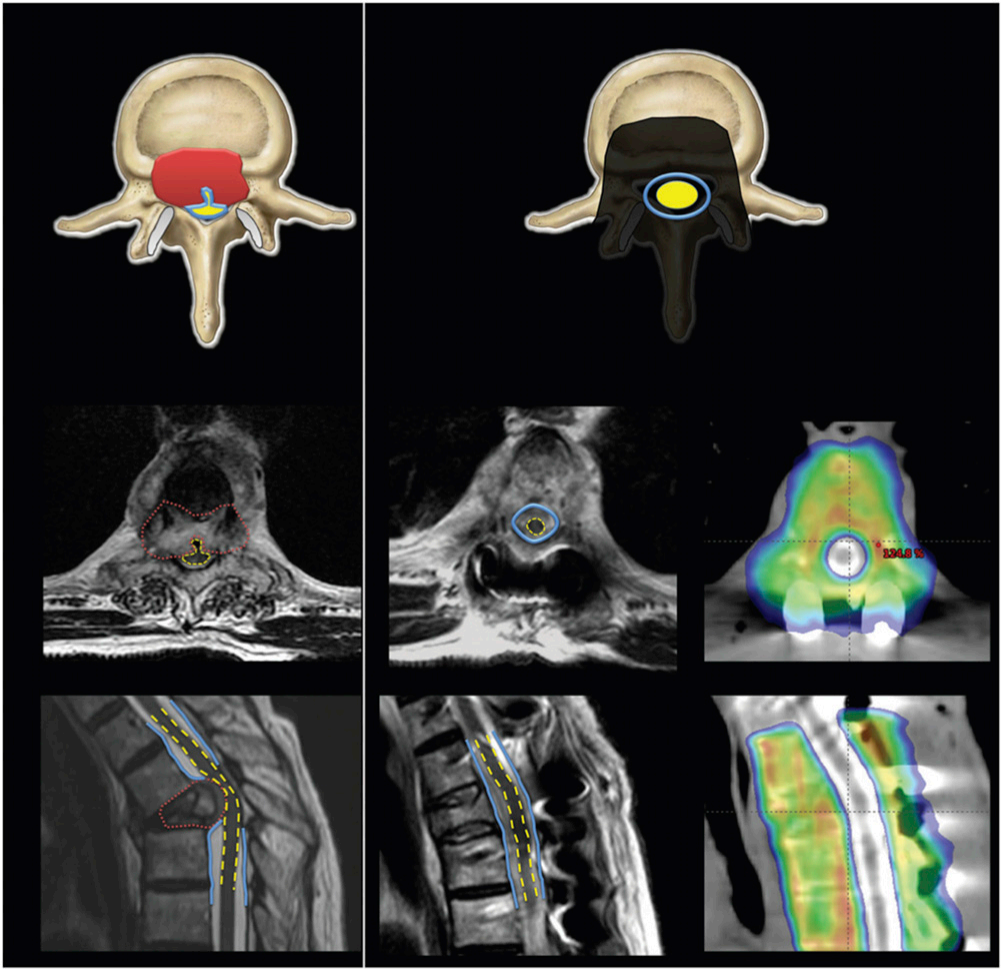
Example of separation surgery followed by stereotactic spine radiotherapy in a patient with spinal cord compression. (A) Pretreatment T2 MRI demonstrates the tumour causing spinal cord compression—the tumour is shown in red, the thecal sac in blue, and the spinal cord in yellow. The sagittal image demonstrates severe cord compression from tumour and bone retropulsion. (B) Postoperative MRI shows decreased canal stenosis and increased separation between the tumour and spinal cord. CT myelogram shows the stereotactic body radiotherapy dose distribution with sparing of the spinal cord. The thecal sac is shown in blue, and the spinal cord in yellow. Reprinted and permission taken from The Lancet Oncology 69.

Bilsky^76^ described the technique of ‘separation surgery’, which entails creating a gap of 2-3 mm between the spinal cord and compressive tumour. Instead of maximal tumour resection to decompress the spinal cord, a minimal epidural decompression is performed, and a plane is created surgically between the tumour and the full circumference of the dura. This then facilitates the creation of a safe zone for SRS to work effectively on residual tumour. Theoretical benefits include less tumour handling, which can reduce surgical time and blood loss. Laufer et al^77^ safely treated 186 patients in this manner. They performed separation surgery for patients with radioresistant tumours, followed by SRS. The local recurrence rate at one year was an encouraging at a low 16.4%. With such encouraging results with SRS, the trend has been to resect just the minimal amount of compressing tumour to create a separation gap of 2-3 mm (Figure 5).

Different approaches have been used on the basis of the spinal level of the lesion, how much bone is involved, and surgeon preference, including the transpedicular approach, costotransversectomy, lateral extra-cavitary approach, transthoracic approach, or retroperitoneal approach. The transpedicular approach is by far the most versatile of these techniques, and can be safely used for a 360-degree decompression, and minimizing the number of procedures patients need.^78^

Conventional radiotherapy utilizes radiation portals involving 1 to 2 beams, which is given typically over 5-10 fractions (eg 30 Gy in 10 fractions). This simple technique does not demand high precision or dose conformity to the tumour.^79^ Its effectiveness is limited by the poor radiation tolerance of the spinal cord; as such high doses to the tumor are not practical. Whereas, SRS is a highly conformal multidirectional technique and sharp dose gradients allow delivery of radiation, in a short and convenient schedule (eg 16 Gy in 1 fraction, or 24 Gy in 2 fractions), even when in close proximity to the spinal cord.^79^

### Palliative Surgery

Palliative surgery should be reserved for patients survival is expected to be 3-6 months. Surgical decision making in this group of patients is perhaps the most complicated. The operative plan is highly individualized according to the clinical presentation and the tumoral configuration. Variability in surgical planning among surgeons can be expected and a more standardized approach could perhaps be beneficial. Several considerations aid the decision making: Presence or absence of neurological compression and assessment of spinal stability, tumor cytoreduction in preparation for Radiotherapy. As opposed to wide marginal and en bloc resection, piece-meal resection or debulking of the tumor is commonly implemented. The surgical approach is dependent on the site of compression: Anterior, posterior or combined. Reconstruction should follow if spinal instability ensues. Staged surgery again should be considered if necessary. Circumferential decompression and reconstruction can be achieved via a single posterior approach and should be tailored to specific guidelines that the literature is unfortunately lacking.

#### A. Cement Augmentation

The most minimally invasive surgical procedure used for spinal stabilization is cement augmentation of a vertebral body. Cement augmentation techniques ie vertebroplasty and kyphoplasty in patients with spinal fractures caused by tumors appears to be effective in reducing pain with relatively few complications.^80^ However, the superiority of one method over the other cannot be determined from the available evidence. These minimally invasive techniques give the advantage of starting adjuvant therapy as soon as 1 week after surgery, compared with adjuvant radiotherapy 1 month or more after traditional open surgery to allow time for adequate wound healing.

Patients who benefit most from stabilization have a SINS Score reflective of potential instability (ie 7-12) and typically have a tumor isolated to the anterior portion of the spine. Patients with substantial posterior involvement of spinal elements, such as the facet joints, are unlikely to get pain relief from this procedure alone.^81,82^ The use of cement augmentation through fenestrated screws is a newer trend that may prove similarly useful for mechanical strengthening of spinal constructs in bone that has been pathologically weakened by tumor.^83,84^

#### B. Minimally Invasive Surgery

Minimally invasive stabilization (MIS) and decompression is performed using working tubes and percutaneous pedicle screws. Initially introduced for degenerative spine diseases, the technique has evolved rapidly since the late nineties. Minimally invasive approaches have been reported to be as successful as open techniques for lumbar decompression with less disruption of surrounding soft tissue structures, reduced intraoperative blood loss, reduced opioid dependence, shorter hospitalization and earlier return to work.^85,86^ The most significant advantage of MIS compared to the conventional open surgical techniques is the faster and better early postsurgical clinical outcome.^86^ Spinal surgical site infection is quoted up to 2% in open surgery^87^ and it has been reduced by nearly 10 times via MIS.^88^ Having established itself in the management of degenerative spinal conditions, MIS is increasingly being recommended for metastatic spine disease due to shorted post-operative recovery of patients.^89-92^

Stabilization can be performed to address spinal instability with or without accompanying decompression. Instrumentation is often depended on for long term stabilization, as there is often a high probability that the spine will not fuse properly around sites of metastases due to large gaps in bony structures, local bone destruction by the tumor, and radiotherapy and/or systemic therapy, which can interfere with the fusion process. A recent data MIS for metastatic disease has been shown to be a safe and effective technique for decompression and stabilization of the spine that may yield improved functional outcomes and quality of life.^93-95^

### Conservative Management

Short-term prognosis is expected in these patients due to disseminated disease and/or extensive spinal involvement, therefore limited surgical intervention is advised. Emergency surgical decompression could potentially be offered in cases where acute neurological deficit develops, but again decision should be individualized. Similarly surgical intervention can be offered in the event of acute deformity secondary to pathological fractures, or intractable pain. The surgical planning should be to treat the abnormalities with the least surgical manipulation possible with aim to control pain and/or improve quality of life for the remaining anticipated survival period. Therefore, collaboration with medical and radiation oncologists is of outmost importance to help guide the orthopedic and neurosurgery spine surgeons.

## Surgical Management Algorithm

**Figure 6.**
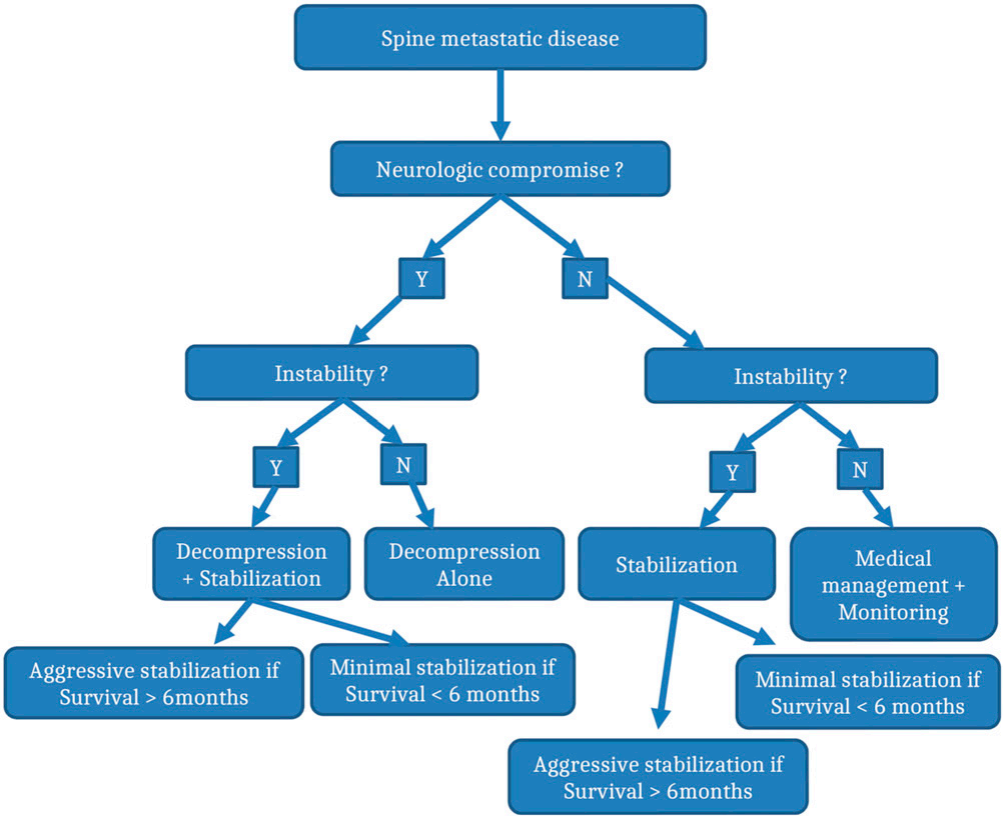
Flowchart synthesized from the best available evidence about the management of metastatic spine. Patients with best projected prognosis should be offered aggressive surgical management to meet those projections. However, poor prognosis warrant more focused approach to relieve pain, restore and/or maintain neurological integrity.

Our flowchart comprehensively encompasses all the relevant, well-studied preoperative assessment scoring systems for patients with spinal metastatic lesions and offer evidence-based surgical planning scheme (Figure 6). We believe it represents the standard with which metastatic spinal lesions should be managed. For any newly diagnosed spinal metastatic lesion(s), we start scoring the case according to prognostic scoring system (eg Tokuhashi or Tomita) which have been shown to be effective in the literature at predicting overall survival,^42,43^ and then looking at spine stability systems (eg SINS). If the patient has good long term prognosis (expected more than 6 months), we consider surgery. In case of solitary metastasis to the spine then En Bloc resection should be considered. If metastatic disease is present in more than one location then intralesional resection or marginal resection should considered with appropriate augmentation to maintain stability.

If stability is compromised as per SINS score then stabilization should be considered in all cases irrespective of expected survivorship. If survivorship is longer than 6 months, then proper stabilization with posterior as well as anterior constructs can be considered. If survivorship is less than 6 months, then one can consider MIS techniques with cement augmentation when appropriate. Other factors to consider are patient overall health status to help guide operative surgical treatment. For example, a very frail patient may better tolerate MIS technique over open technique.

Separation surgery alone should be chosen for those who have canal compromise and no obvious instability that may or may not have neurological compromise in the setting of a radioresistant tumor. If instability is present then augmentation with instrumentation should be chosen based on expected survival (ie more than 6 months can have open stabilization surgery while less than 6 months should be treated with bare minimum to restore stability).

In this paper, we present an algorithm based on survivorship of 6 months or less. We believe that prognosis prediction is one of the limiting factors to spine oncologists and have shown that scores such as Tomita and Tokuhashi are appropriate to predict short term and long term survivorship but are limited and are inaccurate to predict those with intermediate survivorship^42,43^

Therefore, each case with intermediate prognosis should be individualized based on most likely survival according to two broad categories, long term (>6 months) or short term survival (<6 months). If the patient has poor prognosis, palliative surgery or conservative management would be recommended. So if no surgical management indicated, the patient’s care should be centered by medical/radiation oncologists.

## Conclusion

Surgical treatment for spinal metastases has evolved significantly in the past three decades. Landmark studies have provided guidance for patient stratification and surgical planning. In this article we compiled and examined the best available evidence in the literature concerned with spinal metastases and formulated a management flowchart for spinal metastases. The main objective being establishing a standardized approach in the management of these cases. It will also ensure that all new cases will be appropriately stratified and referred for surgical consideration when appropriate. A multidisciplinary approach is highly recommended in the management of these oncological patients. As systemic management of primary tumors continue to improve, the overall survival of patients with metastases disease to the spine is also expected to improve. Revision or establishment of new tumor-specific prognostic scoring systems may come in the near future and help improve prognosis prediction, which is presently the major limiting factor in decision making for these patients. With better improvement it is very likely that more patients will be eligible for surgical intervention in order to improve patient outcomes. Improvement in systemic radiotherapy may also see it become the standard management of selected cases, but for the majority, surgical management remains the standard of care as demonstrated in the literature.
